# Effects of tDCS on Depression and Comorbid Generalized Anxiety Disorder: A Brain Function Imaging Case Report

**DOI:** 10.3389/fneur.2022.879339

**Published:** 2022-06-13

**Authors:** Yuwei Wu, Lin Tang, Xiaolong Shi, Zhiqing Zhou, Yuanli Li, Chunlei Shan

**Affiliations:** ^1^Center of Rehabilitation Medicine, Yueyang Hospital of Integrated Traditional Chinese and Western Medicine, Shanghai, China; ^2^School of Rehabilitation Science, Shanghai University of Traditional Chinese Medicine, Shanghai, China; ^3^Engineering Research Center of Traditional Chinese Medicine Intelligent Rehabilitation, Ministry of Education, Shanghai, China

**Keywords:** transcranial direct current stimulation, generalized anxiety disorder, depression, functional near-infrared spectroscopy, fMRI

## Abstract

**Background:**

Transcranial direct current stimulation (tDCS) is a type of non-invasive brain stimulation technique that has proven effective for neuropsychiatric disorders. Generalized anxiety disorder (GAD) and depression are common psychiatric disorders that often are comorbid, meaning they occur simultaneously. Current evidence supports the value of tDCS for GAD. The objectives of this report is to explore the effect of tDCS on clinical symptoms and cerebral function in a patient with comorbid GAD and depression.

**Methods:**

Our subject was a semiprofessional athlete diagnosed with comorbid GAD and depression. Symptoms included palpitations, sweating, continuous tension, and anxiety. We designed a B-A-B experimental protocol and used the Beck Anxiety Index (BAI), Beck Depression Index (BDI), and Pittsburgh Sleep Quality Index (PSQI) as assessment tools. Treatment consisted of 2 series of 15 days each, separated by a 3-week washout period. We collected functional near-infrared spectroscopy (fNIRS) data before and after both series, as well as fNIRS data immediately after the first treatment in both series. In addition, we collected functional magnetic resonance imaging data before and after the second series.

**Results:**

After the first series, the scores of the three questionnaires (BAI, BDI and PSQI) decreased significantly, which showed the trend of improvement. The functional connection of bilateral prefrontal partial channels decreased significantly immediately after tDCS treatment. The results of the fNIRS before the second-series treatment showed that prefrontal connectivity returned to the state before the first intervention after the washout period. The results of the fNIRS after the second series treatment showed that the symptoms of depression and anxiety alleviated. The results of the fNIRS showed that the prefrontal connectivity decreased again.

**Conclusion:**

In the treatment of comorbid GAD and depression, tDCS can alleviate symptoms and improve sleep quality and social behavior. Brain imaging is widely used to observe functional changes by tDCS such as fMRI and fNIRS. The study also showed that fNIRS can be a safe, simple, and efficient method to assess brain activity.

## Introduction

Generalized anxiety disorder (GAD) and depression are common psychiatric disorders that often are comorbid (1). The Diagnostic and Statistical Manual of Mental Disorders (DSM-IV), published by the American Psychiatric Association, substantially revised the diagnostic criteria for GAD to distinguish it from mood and adjustment disorders, other anxiety disorders, non-pathological anxiety, and other diseases, while it is usually used as an additional diagnosis for other mental illnesses (2). The DSM-V further refined the definition of GAD to include individuals who may have difficulty inhibiting their fears and worries, and who often experience fatigue, difficulty concentrating, irritability, and other symptoms such as muscle tension and sleep disturbance (3). Many studies have shown that anxiety and depression can be lifelong comorbidities (1). For treatment of GAD, psychotherapy is first considered, and psychotropic drugs are also usually recommended to control clinical symptoms of anxiety and depression after diagnosis. However, some patients refuse to adopt psychotherapy or psychotropic drugs. Research has shown non-invasive brain stimulation to be effective and more easily accepted than drugs for psychiatric disorders (4). Transcranial direct current stimulation (tDCS) is a type of non-invasive brain stimulation that regulates the excitability of the cortex by delivering a weak direct current to the brain with two electrodes of opposite polarity (anode and cathode) placed on the scalp (5). Generally, anodic stimulation can excite neurons, whereas cathodic stimulation leads to inhibition (6).

Compared to the side effects of drugs, tDCS is a safer treatment technique. If it is used according to the standard procedures recommended by the latest clinical studies of psychiatric disorders, side effects are rare (5). Common side effects of tDCS include redness, itching, slight tingling, and mild superficial electrolytic burn (7), and these are more acceptable to patients than drug side effects.

## Materials and Methods

### Materials

Our subject was a 33-year-old male semiprofessional athlete who experienced recurring panic attacks and other anxiety symptoms. During an inquiry into his medical history, the patient reported heart discomfort, and the physician ordered a 24-h dynamic electrocardiogram for him. The results showed an average heart rate of 43 beats per minute, with bradycardia as slow as 28 beats per minute at night. Thus, the patient repeatedly worried about sudden death due to bradycardia, and became afraid of his training course. He developed other symptoms such as palpitations, sweating, and tremor, with continuous tension and anxiety during the days. After meeting with a psychiatrist, the patient was diagnosed with GAD, depression, and sleep disorders. The diagnosis of comorbidity was made by psychiatrist according to international Classification of diseases-10(ICD-10). He reportedly worried a lot and had sleep problems due to life pressure and financial pressure. He complained of nightmares, pain, and discomfort during sleep, and reported being easily awakened at night even though he could fall asleep normally. The patient also had a history of alcohol intake. Our patient refused to adopt psychotherapy or psychotropic drugs. It was known that he had treatment with traditional Chinese herbs and acupuncture for his mental illness, but there was no obvious improvement. Thus, he was referred to us for further treatment, and he was willing to try tDCS treatment. The Beck Anxiety Index (BAI), Beck Depression Index (BDI) (8), and Pittsburgh Sleep Quality Index (PSQI) (9) before the first treatment were assessed as 46, 10, and 12, respectively, which classified the patient as having severe anxiety, moderate depression, and normal quality of sleep.

### Methods

**Figure 1:**
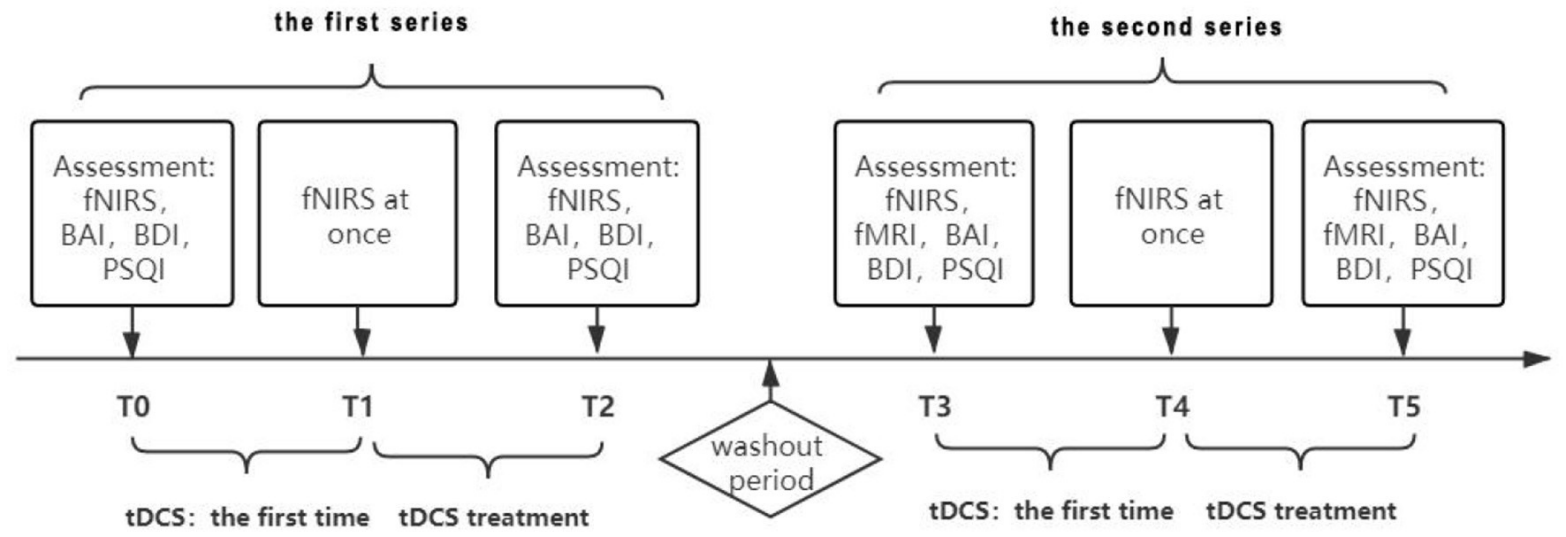
The experimental protocol.

All treatments and data acquisitions were performed in the Yueyang Hospital of Integrated Traditional Chinese and Western Medicine, Shanghai University of Chinese Traditional Medicine. We used a B-A-B design for the treatment protocol (Figure 1). In a single case research, all designs should be attributed to the logic of how to arrange baseline condition (A) and intervention condition (B) (10). The patient may have violent tendencies due to emotional problems, for ethical reasons, we did not collect baseline data in order to avoid potential risks to ourselves and others. Thus, a B-A-B design was adopted, and the experimental sequence was intervention, removal of intervention and re-intervention. The patient accepted the treatment plan after we explained the principles of tDCS. The first intervention series was 15 treatments of tDCS, 5 times a week for 3 weeks, with no intervention on weekends, and this experiment was designed according to another study published in 2014 (11). We used a Volcan model VC-8000F (Nanjing, China) for the treatments. For each 30-min treatment, the anode of the stimulator was placed on the left dorsolateral prefrontal cortex (DLPFC), while the cathode was placed on the right shoulder. During the first treatment with a 1.4 mA current, the patient suffered a mild superficial electrolytic burn, and so we decreased the dose to 1.0 mA. After 1 week, the dose was increased to 1.2 mA. To evaluate the cerebral functional connection of the patient, fNIRS was conducted before and immediately after the first treatment as well as after the last treatment. We also orderd fMRI for the patient, but he refused to complete the evaluation due to fear of the closed environment of the fMRI laboratory. Treatment was suspended for a 3-week washout period between the first and second series. The patient was treated with traditional Chinese medicine and acupuncture during both the treatment series and the washout period.

After the 3-week washout period, the therapists repeated a second series of evaluation and intervention. In this series, we suggested the patient to accept the fMRI. After negotiation with the patient, the patient still expressed fear of the closed environment of the fMRI laboratory, however, the patient told us that he was willing to have a try. This change of his attitude also indicated the improvement of his emotion. This series included functional magnetic resonance imaging (fMRI) scans before the first treatment began and after the last one ended, to explore the effects of tDCS on the subcortical nucleus. As in the first series, the tDCS dose was 1.2 mA and the duration was 30 min. After 15 treatments, we performed fNIRS, fMRI, and behavioral assessments on the patient.

## Results

### Physical Examination

The patient suffered a mild superficial electrolytic burn in the first series of tDCS treatment. There is a 1 ×1 cm-shallow red mark in the forehead of the patient. During the following treatment and follow-up, we paid close attention to the problem of skin lesion, the skin lesion healed in a week and did not appear again in the following treatment. In the follow-up, we also checked the stiuation of the skin lesion and acceptability of tDCS and the patient indicated that he was very likely to accept the tDCS treatment and the side effects. The patient also told us that the skin lesion did not affect his daily life and treatment.

### BAI, BDI, PSQI Scores

After the first series, the BAI score decreased from 46 to 36 and the BDI score decreased from 10 to 9. The total PSQI score decreased from 12 to 7, while the sub-score of sleep quality and disorder declined from 3 to 2, the sub-score of persistence declined from 2 to 1, and the sub-score of daytime dysfunction declined from 3 to 1.

**Figure 2:**
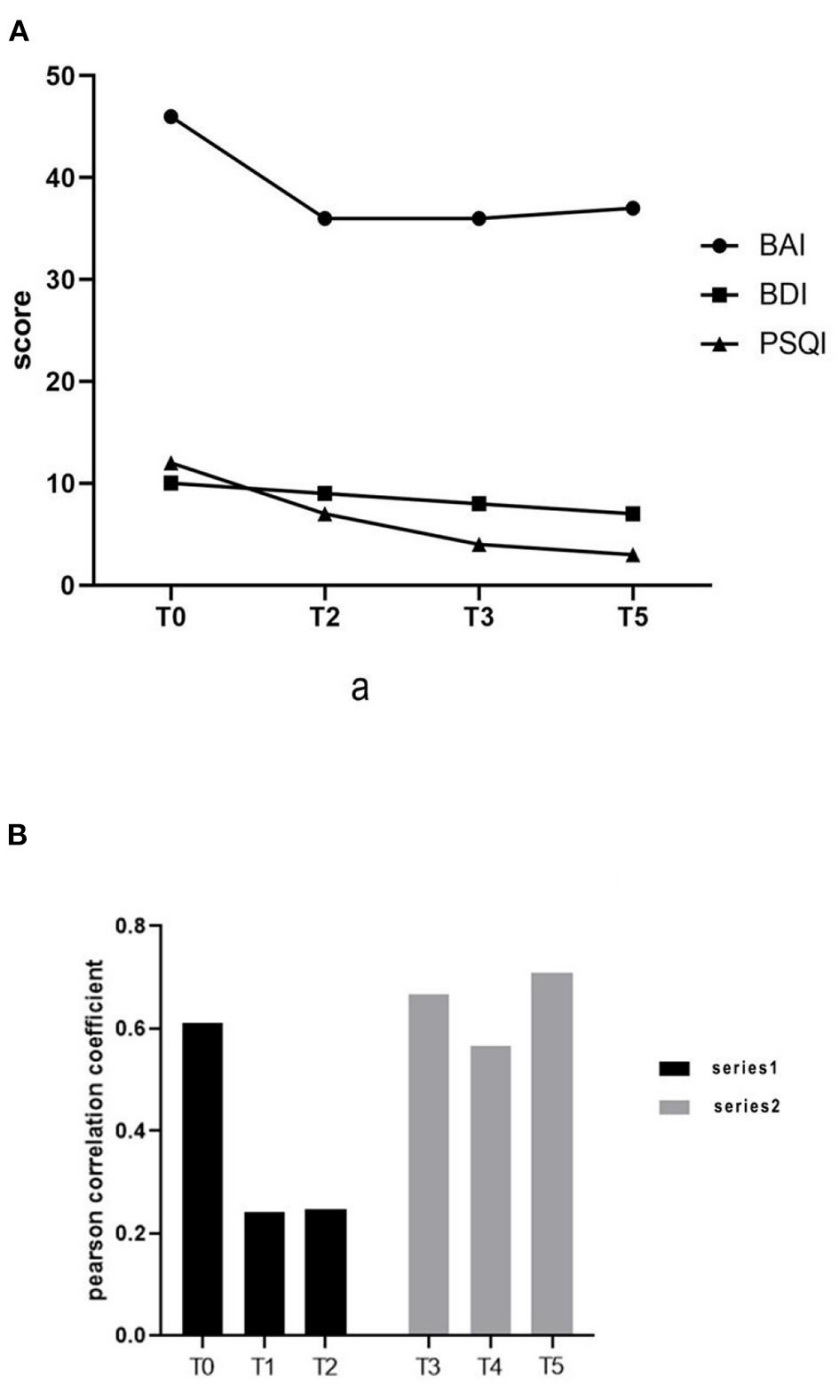
**(A)** Changes in Beck Anxiety Index, Beck Depression Index, and Pittsburgh Sleep Quality Index scores. **(B)** Functional connection of prefrontal cortex.

After the 3-week washout period, we evaluated and intervened again. The pre-intervention evaluation showed that the BAI score was 36, the BDI was 8, and the PSQI score was 4. The sub-scores of sleep quality, sleep disorder, and daytime dysfunction were all decreased by 1 point in the PSQI assessment. After 15 treatments, the results showed a BAI of 37, BDI of 7, and PSQI of 3 (Figure 2A). After an initial decrease, the BAI score remained flat with no significant difference, while the rest of the scores continued to decrease. Sleep problems improved significantly, and the depression level was reduced to the level categorized as “light.”

### fNIRS Data

We collected the fNIRS data with a 45-channel device (NirSmart, Huichuang Medical Equipment, Danyang, China). In the resting state data processing, the Pearson correlation coefficient of oxyhemoglobin on time series of each channel was defined as the resting state functional connection strength between corresponding channels. The fNIRS performed after the washout period and before the first treatment of the second series showed that the patient's prefrontal connection level had returned to the state before the first series (0.66609). The results of the fNIRS immediately after the first intervention of the second series showed that the connection level decreased again (0.56749). At the end of the second series of 15 interventions, the pre-frontal connection level reached the highest state (0.71045) (Figure 2B). This result may have been influenced by external factors such as the patient watching a jiu-jitsu match, or from experiencing fear and anxiety in anticipation of the fMRI test.

**Figure 3:**
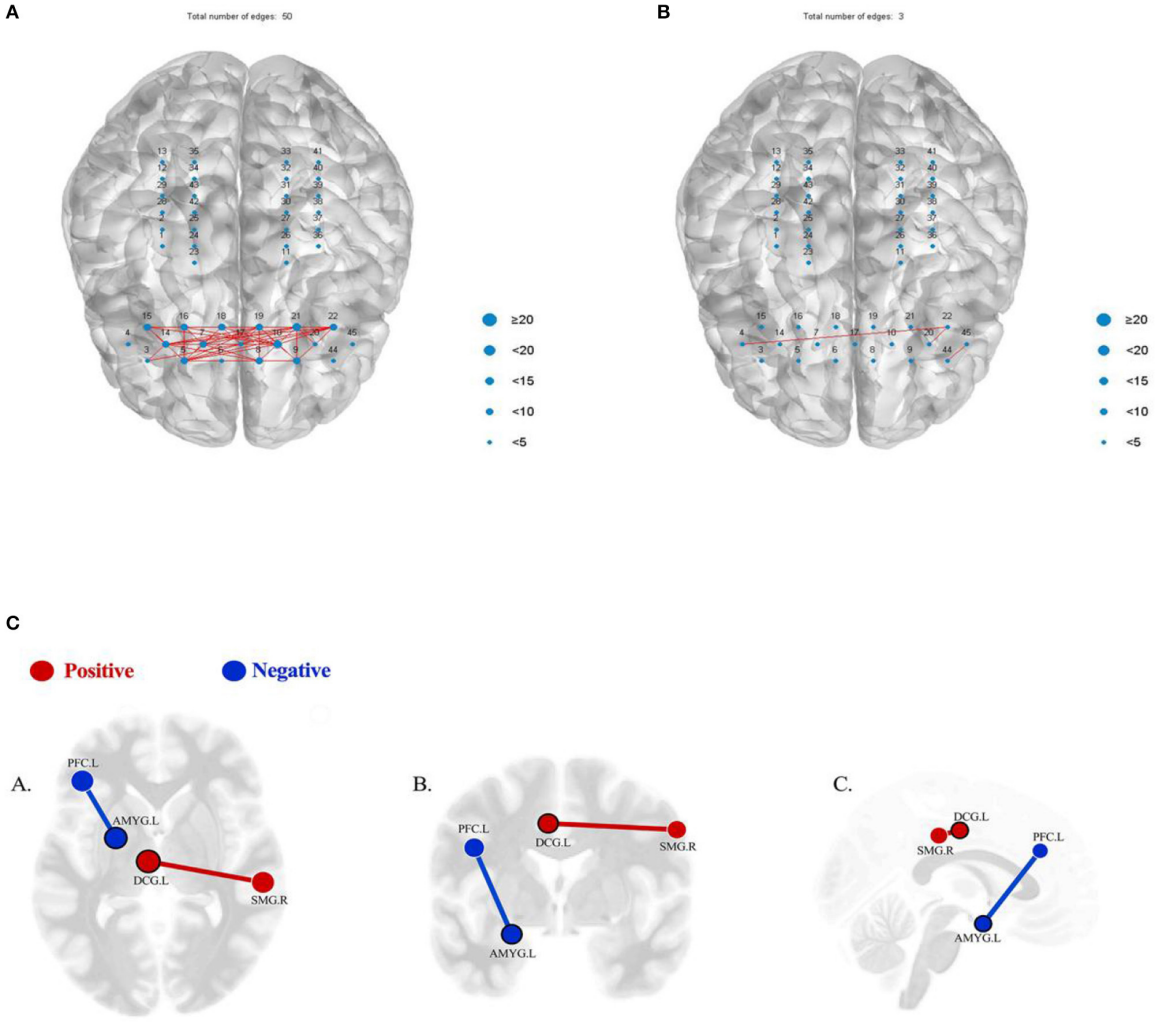
**(A)** When the similarity threshold is 0.8, the functional connection diagram between the prefrontal channels of the patient before the first intervention. **(B)** When the similarity threshold is 0.8, the functional connection diagram between the prefrontal channels immediately after the first intervention. **(C)** Results of seed-based functional connectivity before and after the treatment. Red/positive: the result showing a significant hypo-connectivity between AMYG.L and PFC.L (*p* = 0.0391, FDR corrected), before the treatment>after the treatment; blue/negative: the result showing a significant hyper-connectivity between DCG.L and SMG.R (*p* = 0.0262, FDR corrected), after the treatment>before the treatment (*p* < 0.05, FDR corrected). (A) Axial view; (B) coronal view; (C) sagittal view. AMYG.L, left amygdala; PFC.L, left prefrontal cortex; DCG.L, left median cingulate and paracingulate gyri; SMG.R, right supramarginal gyrus.

The results showed that the functional connection strength of bilateral prefrontal partial channels decreased significantly after tDCS treatment (Figures 3A,B). The mean Pearson correlation coefficient of the prefrontal lobe before the first intervention of the first series was 0.61181. After the first tDCS intervention, it was significantly reduced to 0.24195. Following the complete series of 15 interventions, the mean Pearson correlation coefficient remained at a low level (0.2482). After that, treatment was suspended for a 3-week washout period.

### fMRI Data

The fMRI data acquisition was performed with a 3T MRI scanner (Siemens Verio, Erlangen, Germany). We collected the resting state data of the patient, and all statistical analysis was performed with the CONN toolbox. We used a seed-to-seed analysis to assess differences in connections between brain regions before and after treatment in the second series. False discovery rate (FDR) corrections for multiple comparisons was *p* < 0.05. The seed-to-seed analysis of resting state data showed a significant hypo-connectivity between the left amygdala (AMYG.L) and left prefrontal cortex (PFC.L) (*p* = 0.0391, FDR corrected) and a significant hyper-connectivity between the left median cingulate and paracingulate gyri (DCG.L) and right supramarginal gyrus (SMG.R) (*p* = 0.0262, FDR corrected) (Figure 3C). However, no significant differences were found using the AMYG.L, PFC.L, DCG.L, and SMG.R as seeds in a seed-to-voxel analysis.

## Discussion

In 2014, Shiozawa et al. (11) published a case report of the use of tDCS to treat GAD. Unlike our case, that study used the cathode to treat the right DLPFC, and only the anxiety and depression scale were used for evaluation. The results showed that tDCS did have a significant effect on GAD. A systematic review by Vicario (4) points out that most of the current studies of tDCS intervention on GAD use cathodic inhibition, but that anodic stimulation using tDCS is becoming a trend. Research published by Ana Lucia (12) took into account the comorbidity of depression and anxiety, performed anode stimulation on the left DLPFC, and used the anxiety and depression scale to evaluate the short-term effect of tDCS in the treatment of GAD. This was the first randomized controlled trial to detect the short-term effect of tDCS on GAD, but the effect was not significant due to the relatively short intervention period.

Drawing from the positive results of individual cases and randomized controlled studies, we selected anode intervention on the left DLPFC for 15 days (except weekends) to observe the long-term effects of anode tDCS intervention in our patient with comorbid anxiety and depression. In addition, we also used fNIRS and fMRI to monitor and analyze the brain function. At present, ours is the first study we have found using fNIRS and fMRI data to obtain a more detailed understanding of the effects of tDCS on the brain regulation in GAD.

In our case study, the behavioral assessment showed that tDCS did have an effect on the patient's anxiety symptoms, and the reduction in anxiety lasted for a significant period of time. However, the second series achieved only a maintenance effect and not a diminishing effect. This may have been due to increased anxiety caused by factors in the patient's environment.

Compared with the first series of the treatment, during the second series, the patient's mental symptoms were more stable, and he was motivated to participate in more social activities such as doing high-intensive sports due to improved mood. This increased social activity may have increased his anxiety. For example, before his last two treatments, the patient watched a jiu-jitsu game and left the venue due to discomfort and fear caused by the environment, and reported that it took him 4 h to calm down. The patient's anxiety symptoms might have more significantly improved if he were able to moderate and reduce his participation in situations that increased anxiety. In contrast, through the behavioral evaluation, we observed a steady decline in the patient's depression index and sleep index values. Depression and sleep conditions continued to improve even while the patient was in the washout period. The results support the use of tDCS for patients with depression or sleep disorders. However, the non-specific interventional effects by care-giving and placebo effects by patients' expectations can also contribute to the behavioral changes, which could be relevant confounding factors when the research is based on a single case.

The fNIRS data showed that in the first series, the reduction in prefrontal cortex connection was more obvious than in the second series, both after the first tDCS treatment and at the end of the series. Functional connection values in the first data collection following the washout period increased compared to the values in the first series. We speculated that the increase was due to the suspended tDCS treatment, but we did not rule out mood change caused by increased social activities and the patient's self-reported fear of the fMRI examination in the later period.

The increase in fNIRS connection values after the washout period and the relative reduction after the first intervention indicate that the patient's emotional state was volatile, but the short-term effectiveness of tDCS was evident. The increase in values at the end of the second series showed that adverse external events can have a great impact on patients, which suggests that we should expand the sample size in subsequent studies to minimize the impact of such events.

Although there are certain differences between the results revealed by fNIRS and the behavioral results, we conclude that fNIRS may be a more rapid and effective way to monitor a patient's emotional fluctuations. However, compared with fNIRS, the fMRI results showed no significant difference before and after the second series of treatments. Because fMRI can increase anxiety in the patient, it may not be the best tool to evaluate the patient's emotional fluctuations, but it could still be useful to observe the influence of tDCS and environmental factors on the patient's brain connections.

Our case study showed that tDCS intervention for the patient with GAD comorbid with depression did have some effect, particularly on the patient's depression and sleep conditions. However, after initial improvement, the patient's anxiety level showed no additional progress from the end of the first series through the end of the second. This result may be related to the patient's increased participation in social activities in the later period, or it may be related to the dose. Additionally, the limitation of this case report is lack of quantitative data by means of standardized measures regarding functional outcomes. Therefore, in the future, we may focus on the effects of different doses and exercise conditions on patients. Most of the measures studied here showed major changes during the first week, but not thereafter. This may indicate that a shorter period of stimulation (or smaller number of stimulation) is sufficient to obtain maximum effects of tDCS, which is worthy of further exploration and research in the future. In addition, literature related to GAD intervention (8) mentions that some patients continue using drugs while undergoing tDCS. It may be that in patients who use drugs combined with tDCS, anxiety symptoms can improve more quickly and effectively. In recent studies, we have learned that tDCS does have advantages for mental health, so in the future we may focus on the exploration of multimodal brain functional mechanisms.

## Data Availability Statement

The raw data supporting the conclusions of this article will be made available by the authors, without undue reservation.

## Ethics Statement

Ethical review and approval was not required for the study on human participants in accordance with the local legislation and institutional requirements. The patients/participants provided their written informed consent to participate in this study.

## Author Contributions

YW and LT designed and conceptualized the study, drafted the manuscript, and created the therapeutic intervention. XS and ZZ collected the information and organized the data. YL and CS analyzed the data. All authors approved the final version of the manuscript.

## Funding

This work was supported by the National Natural Science Fund (Grant No. 81874035), the National Key Research and Development Program of China (Grant No. 2018YFC2001600/04), and the Clinical Science and Technology Innovation Project of Shanghai Shen Kang Hospital Development Center (Grant No. SHDC12018126).

## Conflict of Interest

The authors declare that the research was conducted in the absence of any commercial or financial relationships that could be construed as a potential conflict of interest.

## Publisher's Note

All claims expressed in this article are solely those of the authors and do not necessarily represent those of their affiliated organizations, or those of the publisher, the editors and the reviewers. Any product that may be evaluated in this article, or claim that may be made by its manufacturer, is not guaranteed or endorsed by the publisher.
